# Virome-wide detection of natural infection events and the associated antibody dynamics using longitudinal highly-multiplexed serology

**DOI:** 10.1038/s41467-023-37378-z

**Published:** 2023-03-30

**Authors:** Erin J. Kelley, Sierra N. Henson, Fatima Rahee, Annalee S. Boyle, Anna L. Engelbrektson, Georgia A. Nelson, Heather L. Mead, N. Leigh Anderson, Morteza Razavi, Richard Yip, Jason T. Ladner, Thomas J. Scriba, John A. Altin

**Affiliations:** 1 The Translational Genomics Research Institute (TGen), Flagstaff and Phoenix, AZ USA; 2 SISCAPA Assay Technologies, Inc., Washington, DC USA; 3 The Pathogen and Microbiome Institute, Northern Arizona University, Flagstaff, AZ USA; 4 South African Tuberculosis Vaccine Initiative and Institute of Infectious Disease and Molecular Medicine, Division of Immunology, Department of Pathology, University of Cape Town, Cape Town, South Africa

**Keywords:** Viral infection, Statistical methods

## Abstract

Current methods for detecting infections either require a sample collected from an actively infected site, are limited in the number of agents they can query, and/or yield no information on the immune response. Here we present an approach that uses temporally coordinated changes in highly-multiplexed antibody measurements from longitudinal blood samples to monitor infection events at sub-species resolution across the human virome. In a longitudinally-sampled cohort of South African adolescents representing >100 person-years, we identify >650 events across 48 virus species and observe strong epidemic effects, including high-incidence waves of *Aichivirus A* and the D68 subtype of *Enterovirus D* earlier than their widespread circulation was appreciated. In separate cohorts of adults who were sampled at higher frequency using self-collected dried blood spots, we show that such events temporally correlate with symptoms and transient inflammatory biomarker elevations, and observe the responding antibodies to persist for periods ranging from ≤1 week to >5 years. Our approach generates a rich view of viral/host dynamics, supporting novel studies in immunology and epidemiology.

## Abstract (web summary)

Methods to detect infections are often limited to specific viruses or do not yield information on the immune response. Here, the authors analyse temporal changes in high-dimensional antibody profiles and chart more than 650 infection events across the human virome providing a high-resolution view of host-virus dynamics.

## Introduction

Hundreds of species of viruses infect humans (‘human virome’)^1,2^, with consequences ranging from asymptomatic seroconversion to severe disease, which can include respiratory failure, neurological damage or hemorrhage. During a typical year, the average person becomes naturally infected with multiple species^3^, some asymptomatically^4^, but most leaving an immunological footprint that can continue to evolve months after the infection^5^. In addition to their pathogenic properties, naturally-infecting viruses can be considered as a set of natural perturbations to the immune system; and the responses against them as reporters of the host’s current ‘immunological state’.

The most common approaches for detecting viral infections, particularly in clinical settings, rely on the direct detection of proteins or nucleic acid sequences of the infecting agent. Measurements of this type can be extended to multiple simultaneous targets (e.g. multiplexed PCR)^6^, or implemented in a target-agnostic mode using deep DNA/RNA sequencing and metagenomic analysis^7,8^. While such direct approaches can detect pathogens early in the course of infection, they are fundamentally limited by a requirement that the sample contain the infectious agent—substantially constraining the body site and timing at which sensitive detection can be performed. Serological approaches, by contrast, rely on an immune response whose effect is to amplify, sustain and disseminate the signal, allowing infections to be inferred from blood samples collected months after the event. Epidemiological studies have leveraged these properties to great effect, for example in the estimation of incidences of particular viral infections within populations^9^, or of the duration of protection from re-infection^10^. Relative to direct detection by metagenomics, however, serological assays have traditionally been limited in the number of agents they can query simultaneously, as well as their ability to resolve between closely-related pathogens.

Assays that measure antibodies against single targets (e.g. ELISA) are incommensurate with the vast antigenic space encountered by the human immune system. This limitation is being surmounted by high-dimensional approaches based on arrays of proteins/peptides^11^, or libraries of DNA-associated peptides, either displayed on phage particles (PhiP-Seq^12,13^), or in the form of direct peptide:DNA conjugates (PepSeq^14–16^). Although these assays have enabled reactivity to be profiled across broad target spaces—including the complete human virome—important challenges remain, including resolving contemporary from historical infection events, and accurately distinguishing signal from noise in high-dimensional datasets. The analysis of temporally-separated samples from the same donor has the potential to address these challenges, by enabling normalization to participant-specific baselines against which responses can be more confidently detected and localized in time.

Recent studies have begun to apply highly-multiplexed antibody assays to longitudinally-sampled cohorts^17^, including studies that have identified changing viral reactivity profiles following kidney^18^ and hematopoietic cell^19^ transplantation, as well as a striking association between Epstein-Barr Virus seroconversion and the development of Multiple Sclerosis^20^. However, methods for identifying time-resolved species-level infection events across the virome are needed. Moreover, virome-wide assays have not been used to follow the dynamics of antibody responses to natural infections or to determine how such signals associate with independent indicators of infection such as inflammatory or epidemic events. In this study, we combine a highly-multiplexed virome-wide antibody assay (using the PepSeq platform) with an innovative approach for the detection and monitoring of virus-specific temporal changes across the resulting high-dimensional, time-resolved data. We apply these methods to study antibody responses across several longitudinally-sampled cohorts, including: (i) a spatiotemporally-linked cohort of South African adolescents spanning >100 person-years in which we study population-level effects, and (ii) separate, smaller cohorts of more frequently-sampled participants in which we track antibody dynamics alongside orthogonal markers of infection in individual participants.

## Results

### Quantifying species-specific temporal changes in virome-wide PepSeq data

To study the dynamics of antibody responses at high-resolution across the virome, we used the PepSeq platform (assay scheme outlined in Supplementary Fig. 1) in which 1000s–100,000s of DNA-barcoded peptides are prepared from fully-programmable oligonucleotide templates using bulk enzymatic reactions, and then used to measure antibody reactivity in polyclonal serum samples at high multiplexity^14–16^. The HV1 PepSeq library has been previously described^14^ and consists of 244,000 30mer peptides representing the proteomes of several hundred human-infecting viral species. In addition to HV1, we used empirically-refined libraries (‘HV2’, ‘HV2T’) each comprising 15,000 peptides representing 80 common human-infecting virus species (Supplementary Data 1), and selected on the basis of previous assays with HV1 (see Methods). Consistent with our previous studies, we found PepSeq to recapitulate the expected reactivities for viral species at single timepoints in control samples with known historical exposure status, and to yield consistent peptide signals across runs (Supplementary Fig. 2).

**Fig. 1:**
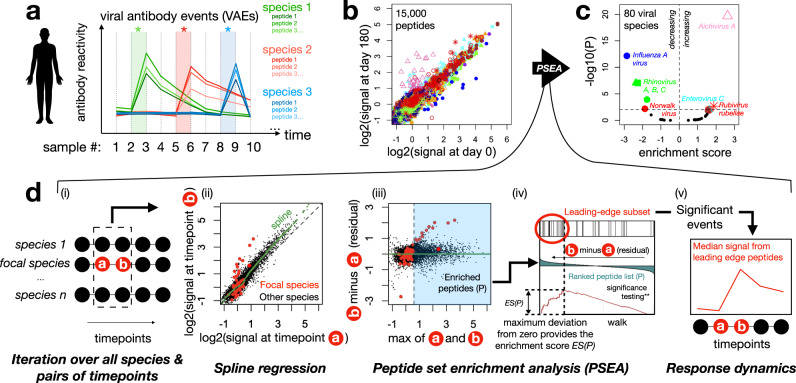
Virus-specific events inferred from co-ordinated temporal changes in highly-multiplexed antibody reactivity data. **a** Conceptual figure showing how viral events—corresponding to infections or reactivations—appear in a longitudinal series of multiplexed antibody reactivity data. **b**, **c** Example plots showing the transformation of peptide-level signals (**b**) into species-level signals (**c**) from two consecutive timepoints from an individual participant, separated by 180 days: (**b**) shows the magnitude of reactivity at each timepoint against 15,000 30mer peptides in the HV2T human virome library, each peptide is represented by a dot whose color and shape reflect its species of origin, while (**c**) shows the change in species-level reactivity to each of the 80 virus species covered in HV2T, expressed as an Enrichment Score (x-axis: positive=increasing over time, negative = decreasing over time) and adjusted *p* value (y-axis). The data shown in (**c**) were calculated using the algorithm described in (**d**). Species with adjusted *p* < 0.01 are highlighted, using symbols matching (**b**). **d** To detect viral events, our algorithm considers each pair of consecutive samples (“**a**” and “**b**”) in a longitudinal series and tests for evidence of differential signal for each virus across that interval (i). First, a spline (green line) is fitted to peptide-level data from the focal timepoints to regress out global differences (assumed to be technical rather than biological (ii)). For the subset of peptides (P, blue shading) whose maximum signal across the two timepoints exceeds a threshold (vertical line in (iii)), spline residuals (representing the magnitude of the change) are fed into the Gene Set Enrichment Analysis (21), which we here refer to as Peptide Set Enrichment Analysis ‘PSEA’. For each species, PSEA iterates down the ordered list of residuals for P and increments a walk function according to the magnitude of the residual—up if the peptide corresponds to the focal species, and down if not (iv—adapted from (21)). Maximum deviation of the walk from zero is used to determine significance, the Enrichment Score, as well as a “leading-edge” subset of peptides that can be used to visualize response dynamics across the time series (v).

To resolve new viral infections from historical exposures, and identify the peptides bound by the responding antibodies, we developed a method for quantifying changes in the reactivity towards each viral species in consecutive longitudinal samples from the same participant (Fig. 1). Peptide-level enrichment signals were strongly correlated between timepoints for the same participant, dominated by a majority of peptides that were unenriched at both timepoints (Fig. 1b: lower-left region) and a subset of peptides with various degrees of enrichment that were consistent between timepoints (Fig. 1b: upper-right region). In some cases, however, we observed additional subsets of peptides with differential reactivity between timepoints (Fig. 1b: upper-left/lower-right regions): in contrast to the time-invariant enriched peptides, these time-variant enriched peptides were typically strongly concentrated in a single or small number of viral species.

We found that threshold-based approaches were often unable to detect, without substantial loss of specificity, subsets of peptides that clearly appeared to be part of virus-specific time-variant responses, and therefore we developed an approach that could more comprehensively integrate both the number and magnitudes of changing peptide signals for each virus (Fig. 1c, d). Gene Set Enrichment Analysis (GSEA) is a widely-used method for differential gene expression analysis in which the magnitude and ranks of a functionally-related subset of genes are integrated (against a background of non-focal genes) to derive scores reflecting the statistical confidence and magnitude with which the focal gene set is enriched^21^. For example, GSEA has been used to associate p53 deficiency with transcriptional upregulation of a gene module within the mitogen-activated protein kinase pathway in tumor cell lines^21^. We developed ‘PSEA’, an implementation of GSEA in which we analyze peptide sets designed from particular viral species (in the place of gene sets corresponding to particular pathways or functions). In place of the log2 fold change scores typically used in GSEA, we used log2 spline-adjusted changes in PepSeq-based peptide enrichments between consecutive timepoints from the same participant. PSEA outputs *p* values and Enrichment Scores that quantify the differential antibody reactivity to a particular virus species across a focal time interval, as well as a “leading-edge” subset that can be interpreted as the core set of peptides that are recognized by the changing antibody response.

We benchmarked the performance of PSEA for accurately detecting virus-specific responses in longitudinal PepSeq data in a controlled setting by studying a COVID-19 vaccination cohort^15^. Plasma samples were collected from 21 participants at days 0, 8, ~28 and ~140 relative to the first dose of the mRNA-1273 vaccine (Supplementary Data 2) and assayed in duplicate for virome-wide reactivity using the HV2 PepSeq library. On the resulting data, we used PSEA to perform two categories of comparisons: (i) as ‘expected negatives’, we compared reactivity to all 80 species between pairs of replicate assays from the same participant/timepoint and (ii) as ‘expected positives’, we compared reactivity to SARS-CoV-2 (one of the 80 species in the library) between the samples collected on day 0 v day 140 for each participant. At an adjusted *p* value threshold of 0.01, PSEA-detected significant changes in 20/21 (95.2%) of the ‘expected positive’ comparisons, and 12/5360 (0.2%) of the ‘expected negative’ comparisons. As expected, leading-edge peptides for each of the 20 significant ‘expected positive’ comparisons were exclusively derived from the Spike protein, corresponding to the vaccine immunogen. Analysis across the full *p* value range using Receiver-Operating Characteristic analysis yielded an area under the curve of 0.997 (Supplementary Fig. 3). These data indicate that analysis of virome-wide PepSeq data using PSEA enables sensitive and specific detection of a virus-specific antibody response induced by vaccination.

### Virome-wide antibody dynamics in a large, longitudinally-sampled cohort

The Adolescent Cohort Study (ACS) was conducted during 2006–2008 and has been described previously^22^. The ACS enrolled and followed 12–18-year-old *Mycobacterium tuberculosis*-infected participants who attended high schools in the town of Worcester, in the Western Cape of South Africa for a total of 24 months, and included the collection of blood samples at 6-monthly intervals spanning up to 18 months (i.e., four longitudinal samples). For this study, we focused on a subset of 65 participants for whom the full set of 4 × 6-monthly blood samples were available, which includes 8 participants who progressed to active tuberculosis disease during the study period (sampling scheme shown in Supplementary Fig. 4a and Supplementary Data 3). We performed assays using the HV2T PepSeq library, and by applying unsupervised clustering, found that global patterns of signal across the 260 samples were strongly driven by participant of origin—such that all timepoints from a given participant were tightly co-clustered—but without any grouping by timepoint, sex, age or disease progression status (Supplementary Fig. 5).

We next used PSEA to compare all pairs of consecutive samples for all 80 viral species covered—a total of 15,600 temporal species comparisons (65 participants X 3 consecutive sample pairs or “intervals” X 80 viral species). Of these, we found 941 (~6%) to be significant by PSEA, comprising 509 in which species-specific antibodies increased over time and 432 in which they decreased. In many cases, an interval of significantly increasing reactivity within a participant was followed in the one or two subsequent intervals by significantly decreasing reactivity for the same species, consistent with the expected kinetics of an antibody response to infection. To restrict our analysis to unique viral episodes, we therefore focused only on observations of significantly increasing reactivity in intervals 1 (timepoints 1–2), 2 (timepoints 2–3) or 3 (timepoints 3–4), or significantly *decreasing* reactivity in interval 1. We refer to such observations as ‘viral antibody events’ (VAEs), and localize them to the intervals in which the reactivity is increasing, or in the case of observations of decreasing reactivity in interval 1 — to a time preceding interval 1 (“interval 0”).

**Fig. 2:**
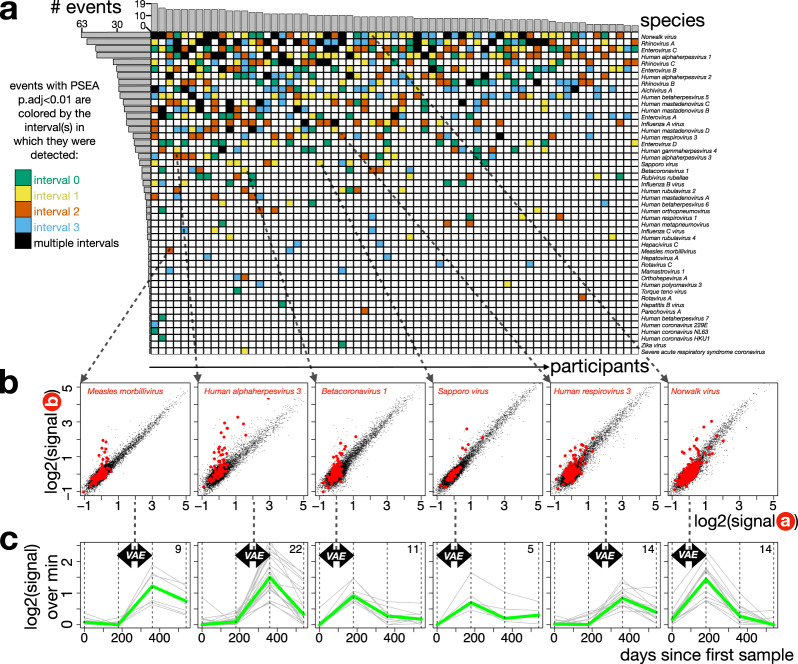
Detection of events across the virome in a cohort spanning ~100 total person-years. **a** For all 65 participants (columns) in the ACS cohort for whom data on 4 × 6-monthly timepoints were available, PSEA analysis was applied to each of the 3 pairs of consecutive timepoints across all 80 viral species represented in the HV2T library. Shown in rows are the 48 species for which ≥1 significant viral event (adjusted *p* value < 0.01) was observed across the cohort. Significant events are colored by the interval(s) in which they were detected. **b** Example peptide-level scatterplots for 6 of the (659 total) viral events identified in (**a**). Each plot shows signals from the 15,000 HV2T peptides (1 dot per peptide) at the 2 timepoints spanning the focal event (x and y-axes), with all peptides from the focal species highlighted in red. **c** Shown for each of the example events in (**b**) is the baseline-subtracted reactivity signal (y-axis) across all 4 timepoints (x-axis) of the leading-edge peptides from the focal virus (each peptide represented as a gray line). Black arrows highlight the interval in which PSEA-detected the significant VAE (from which the leading-edge peptides were identified), and the bolded green line shows the median signal across all leading-edge peptides. Numbers of peptides in each leading-edge set are indicated in the upper right of each plot. Source data are provided as a Source Data file.

Enumerated in this way, a total of 659 VAEs were detected across 48 viral species (1–63 events total per species) and in all 65 participants (4–19 events per participant) (Fig. 2a). Overall, this represents an average of ~5 VAEs per person-year, which we expect to be a lower bound estimate for the true number of events, as our sampling scheme would be insensitive to cases where reactivity rises and falls back to baseline within a single 6-month interval. We detected a number of cases where ≥2 VAEs from the same virus were detected sequentially in the same participant (black cells in Fig. 2a), particularly for species with large numbers of VAEs. For several species, these ‘repeat VAEs’ occurred less often than expected by chance, which may reflect immune-mediated protection (Supplementary Fig. 6). Across all observed VAEs, we detected 2–190 (median = 31) leading-edge peptides (defined as described above) to which antibody reactivity changed, and these typically represented at least two distinct epitopes from the focal species (studied further below). Leading-edge peptides (listed in Supplementary Data 4) showed a similar amino acid composition to unselected viral proteomes (Supplementary Fig. 7a), and included both known and novel epitope sequences (Supplementary Fig. 7b).

Species with the most frequently-occurring VAEs (Fig. 2a, upper rows) were strongly enriched in two categories: (i) widely-circulating acutely-infecting viruses—including *Norwalk virus* (Norovirus) (63 VAEs), *Influenza A virus* (22 VAEs), multiple species of Rhinoviruses (58, 34, and 29 VAEs for *Rhinovirus A*, *C* and *B*, respectively), Enteroviruses (51, 31, 22, and 15 for *Enterovirus C*, *B*, *A* and *D* respectively) and Adenoviruses (25, 24, 20, and 5 for *Human Mastadenovirus C*, *B*, *D* and *A*, respectively); and (ii) latently-infecting Human Herpesviruses (HHVs)—namely, the Herpes Simplex Viruses (49 and 30 VAEs for *Human alphaherpesviruses 1* and *2*, respectively) and Cytomegalovirus (28 VAEs for *Human betaherpesvirus 5*). Since near-universal reactivity was detected for these 3 HHV species at baseline across the cohort, this latter category of events is likely predominantly explained by viral reactivations, which can occur frequently and subclinically, and are often accompanied by elevations in virus-specific antibodies^23^. We also detected VAEs for 4 other HHVs, although at lower rates: 15 for *Human gammaherpesvirus 4*/Epstein-Barr Virus, 11 for *Human alphaherpesvirus 3*/Varicella Zoster Virus, 4 for *Human betaherpesvirus 6*, and 1 for *Human betaherpesvirus 7*, indicating that such events are a common feature of this viral family.

We also observed VAEs for other common respiratory viruses, including the remaining Influenza species (7 for *Influenza B virus*, 3 for *Influenza C virus*), the 4 Parainfluenza species (16 and 4 for *Human Respirovirus 3* and *1*; 6 and 3 for *Human Rubulavirus 2* and *4*), the 4 endemic coronaviruses (8 for *Betacoronavirus 1*/HCoV-OC43, 1 for *HCoV-HKU1*, 1 for *HCoV-229E*, 1 for *HCoV-NL63*), *Human metapneumovirus* (3), and *Human* o*rthopneumovirus* (4). A single VAE was called for *Severe acute respiratory syndrome coronavirus*, a species included in the library but whose major human spillover in 2019 post-dates this cohort. However, this event exclusively involved an epitope known to be cross-reactive with HCoV-OC43^14^ and was observed to precisely coincide with a HCoV-OC43 VAE that included additional OC43-specific reactivities. Beyond *Norwalk virus*, VAEs for a number of agents of acute gastroenteritis were also detected, including 29 for *Aichivirus A*, 10 for *Sapporo virus*, and 3 for the Rotavirus species. Also notable is the observation of sporadic VAEs for viruses that are usually targeted by childhood vaccination (8 for *Rubivirus rubellae*, 2 for *Measles morbillivirus*), as well as 1 for Zika Virus.

Comparisons of the overall numbers of VAEs across participant covariate categories (Supplementary Fig. 8) revealed significantly fewer VAEs among participants who progressed to active tuberculosis (median 5.5, compared to 10 in non-progressors). We also observed more VAEs in males (median 13 VAEs per participant, compared to 9 in females). The absence of corresponding deviations in the number of leading-edge peptides per VAE across these categories indicates that the VAE count differences are unlikely to reflect biases in antibody-based detection, and instead suggests differential rates of exposure/susceptibility to infection, which in the case of sex, is consistent with previous reports for particular species^24^.

In addition to pairwise comparisons of consecutive timepoints, we studied species-specific dynamics across all 4 timepoints (Fig. 2b, c). To accomplish this, we began with VAEs detected using pairwise PSEA comparisons as above (Fig. 2b shows 6 examples), and then tracked the reactivity signals of the leading-edge peptides across the full time series (Fig. 2c). To avoid the subtle but global sample-to-sample signal differences mentioned earlier, we generalized the spline approach to adjust data from each timepoint against the peptide-wise median signals across all timepoints. We then focused the visualization on changing reactivities, as opposed to absolute values, by normalizing each peptide against its minimal value across the time series. The dynamics of leading-edge peptide reactivities typically comprised sharp single-interval increases followed by more gradual declines, matching the expected kinetics of an immune response. For most VAEs, antibodies remained elevated above baseline for the remainder of the sampling period – often ≥6–12 months after the VAE interval. The observation of these classic response kinetics beyond the intervals with increasing reactivity lends further support to our interpretation of PSEA-detected VAEs as viral infection or reactivation events.

To compare our PepSeq-based PSEA analysis against a more traditional serological approach, we selected two species for which we detected frequent VAEs and where commercial ELISA assays are available: *Influenza A virus* and *Human betaherpesvirus 5* (CMV). For each, we selected pairs of consecutive samples that either contained or did not contain a VAE and assayed each using the corresponding ELISAs. The magnitudes of temporal changes in ELISA reactivity across each time interval were strongly correlated with PepSeq VAE status for both of the viral species studied (Supplementary Fig. 9), indicating a high degree of concordance between our multiplexed peptide-based approach and traditional singleplex assays based on native viruses that contain full-length proteins.

### Detection of epidemiological effects for a subset of viruses

**Fig. 3:**
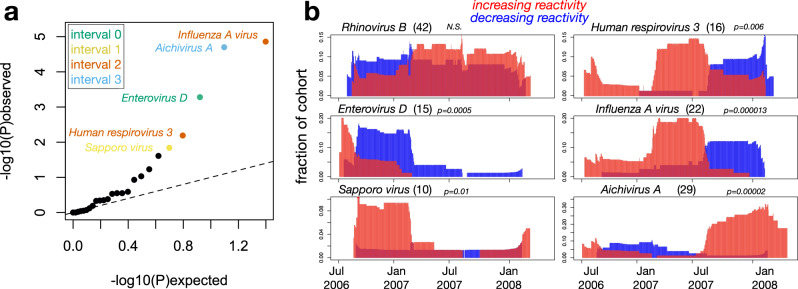
Seasonality analysis reveals epidemic waves for a subset of viral species. **a** For each species, the viral events detected across the cohort described in Fig. 2 were localized to one of the sampling 6-monthly intervals, and seasonal effects were quantified by comparing the observed distributions against a one-sided multinomial model in which events were distributed across intervals with equal probability. Shown is a quantile-quantile plot of unadjusted −log10(probabilities) for each of 25 species for which ≥5 total events were observed across the cohort. The five species with FDR-adjusted *p* values < 0.1 are highlighted and colored according to the interval in which their event count is maximum. **b** For the five significant species identified in (**a**), and *Rhinovirus B* as a negative control, profiles of cohort-wide reactivity dynamics were generated by assigning to each day in the sampling period (Jul 12 2006–May 26 2008) scores representing the number of participants for whom reactivity was significantly increasing (red) or decreasing (blue), normalized to the total number of participants with samples spanning that day. Increasing/decreasing reactivity assignments were based on significant PSEA values and applied in each case to every day between the 2 relevant consecutive sampling dates. Each plot is annotated with the species name, the total number of VAEs detected (in parentheses), and the u\nadjusted one-sided multinomial *p* value calculated in (**a**). Source data are provided as a Source Data file.

Because the ACS cohort comprises participants sampled approximately synchronously (with their 4-sample series beginning over a 5 month window from July–November 2006) and who reside in the same geographic region, we reasoned that naturally-occuring epidemic waves may be detectable in our viral antibody analysis as clusters of temporally-related VAEs. To test this hypothesis, we focused on the 25 species for which we detected ≥5 VAEs across the cohort, localized these events into one of the 4 intervals described above (interval 0, interval 1, interval 2, interval 3; corresponding, respectively, to pre-Aug 2006, Aug 2006–Feb 2007, Feb 2007–Aug 2007, and Aug 2007–Feb 2008) and used a null model in which events are distributed across intervals with equal probability to estimate the significance of the observed patterns. Applying this approach, we observed strong evidence of epidemic effects across the dataset (Fig. 3a): 6 of 25 (24%) species yielded *p* values < 0.05, of which 5 remained significant after adjusting for multiple testing at FDR < 0.1 (Fig. 3a: highlighted datapoints, colored by season). *Influenza A virus* showed the strongest evidence of an epidemic effect, with 16 of 22 total VAEs clustered in interval 2, which spans the Southern Hemisphere winter season of 2007. Other significant epidemic waves included *Aichivirus A* (19 of 29 VAEs observed in interval 4) and *Enterovirus D* (11 of 15 VAEs observed in interval 0).

To visualize cohort-wide virus-specific antibody changes in both directions and more continuously over the sampling period, we quantified aggregate changes in reactivity (increasing and decreasing) across the cohort for each of the viruses exhibiting significant epidemic effects. For each calendar day, we enumerated participants with increasing v decreasing reactivity (measured by PSEA) to the focal virus in samples spanning that day and normalized to the total number of individuals with spanning samples, thereby estimating the fraction of the cohort with increasing or decreasing reactivity to the virus at each point in time (Fig. 3b). On this view, the epidemic viruses generally conformed to a stereotypical pattern consisting of a wave of increasing reactivity, followed by an approximately symmetric wave of decreasing reactivity, offset by 1 sampling interval (equivalent to ~6 months). For *Aichivirus A*, the wave of increasing reactivity occurred at the end of the overall sampling period, meaning that the predicted subsequent wave of decreasing reactivity was not captured in the sampling window. Similarly, the strong wave of decreasing reactivity to *Enterovirus D* (peaking ~Aug 2006–Feb 2007) was preceded by a smaller degree of increasing reactivity (peaking ~July 2006), likely representing a truncation of a wave predicted to localize prior to the beginning of the study in July 2006 (“interval 0”). At their peak, the biggest waves—*Influenza A virus*, *Aichivirus A* and *Enterovirus D*—affected 20–30% of the cohort, indicating relatively high incidences of infection. Together, these results demonstrate virome-wide detection of epidemic waves, manifesting as species-specific seasonally-correlated antibody dynamics within a spatiotemporally-localized cohort.

### Sub-species resolution of viral antibody events using epitope-level analysis

One of the strengths of direct sequence-based viral detection is the ability to resolve between closely-related subtypes by means of sequence polymorphisms. Serological approaches, by contrast, provide an indirect link to viral sequences and are complicated by the potential for cross-reactive antibody responses against (often immunodominant) regions conserved between members of the same species. We reasoned that our peptide-resolved approach may nonetheless be capable of sub-species resolution, insofar as it enables responses against subtype-specific epitopes to be dissected from confounding species-wide responses against conserved epitopes. We set out to test this hypothesis using epitope-level characterization of the antibody responses to several of the epidemic viral waves identified above.

**Fig. 4:**
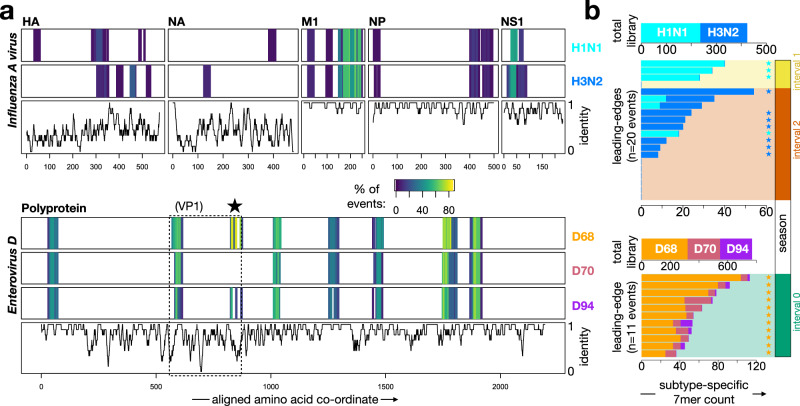
Epitope-level analysis resolves *Influenza A virus* and *Enterovirus D* epidemic waves to the sub-species level. **a** Leading-edge peptides from the events detected in Fig. 2 for *Influenza A virus* (*upper*) and *Enterovirus D* (*lower*) were mapped onto representative viral proteomes of the dominant respective circulating subtypes (*Influenza A virus*—H1N1, H3N2; *Enterovirus D*—D68, D70, D94). Shown in the upper rows of each panel are multiple sequence alignments for the reactive proteins of each virus, colored at each position by the fraction of total events whose leading-edge peptides contain the 7mer at that position for each subtype. The lower row of each panel shows average amino acid identity between the subtype representatives across a sliding window of 9 amino acids centered at each amino acid position in the alignment. The dotted box in the *Enterovirus D* panel shows the position of the surface protein VP1, and the star indicates a frequently-recognized variable region containing D68-specific reactivity. **b** To resolve the subtype identity of each *Influenza A virus* and *Enterovirus D* event, sets of subtype-specific 7mers were quantified for each virus/subtype—both in the total library (top of each panel), and in the leading-edge set of peptides for each event (body of each panel). Subtype-specific 7mers were present for 13/20 *Influenza A virus* events and 11/11 *Enterovirus D* events; for the remaining events leading-edge peptides contained only 7mers shared between subtypes. For each event, the significance of its subtype assignment was determined by calculating the deviation of the event’s subtype-specific 7mer distribution from the subtype-specific 7mer distribution in the total library (using two-sided Fisher’s exact test): stars indicate events with *p* < 0.01. The season corresponding to each event is indicated on the far-right. Source data are provided as a Source Data file.

We selected *Influenza A virus* and *Enterovirus D* as two clinically-significant virus species with well-described circulating subtypes, and as the agents of two of the three largest epidemic waves observed in this cohort. We began our analysis by mapping all 7mer amino acid substrings of leading-edge peptides in the VAEs for each species to any identical substrings within representative proteomes of the dominant circulating subtypes: H1N1 and H3N2 for *Influenza A virus*, and D68, D70 and D94 for *Enterovirus D* (Fig. 4a). Using this approach, we mapped reactivity to linear epitopes across 5 of the 11 proteins of *Influenza A virus*: Hemagglutinin (HA), Neuraminidase (NA), Matrix 1 (M1), Nucleoprotein (NP) and Non-structural protein 1 (NS1) (Fig. 4a, upper panels). This included prevalent (65-70% of VAEs) but non-subtype-resolving responses against highly-conserved regions of M1 and NS1. Reactivity to NS1, a non-structural component absent from available influenza vaccine preparations^25,26^, confirms the origin of these responses to be natural infection rather than vaccination. Importantly, we also observed responses (albeit less prevalent: each ranging from ~5–25% of VAEs) against 12 highly-divergent epitope regions in the non-conserved HA and NA proteins, providing a basis for subtype discrimination. A similar overall phenomenon was observed for *Enterovirus D*: most epitopes resided in conserved regions across the Polyprotein whose reactivity profiles were correlated across the D68, D70 and D94 subtypes (Fig. 4a, lower panels), but a subset of subtype-specific epitopes were detectable in the region corresponding to the variable surface protein VP1. Notably, we detected a highly-prevalent epitope (82% of VAEs) at the C-terminus of VP1 (marked by a star above the lower panels of Fig. 4a), corresponding to a previously-described D68-specific reactive region^27^.

To integrate epitope signals into sub-species-level calls, we enumerated 7mers specific for each subtype from the leading-edge of each VAE and compared these counts to the subtype-specific 7mer distribution from the overall 15,000-member library (Fig. 4b). For *Influenza A virus* and *Enterovirus D*, totals of 423 and 769 subtype-specific 7mers were present in the overall library, and these were distributed approximately uniformly across the respective subtypes: H1N1 v H3N2 or D68 v D70 v D94 (Fig. 4b, top of each panel). Subtype-specific 7mers were also present (but at lower counts: ranging from 8-116) in the leading-edge peptide sets for 13 of 20 *Influenza A virus* and 11 of 11 *Enterovirus D* events. However, in contrast to their uniform representations in the overall library, these leading-edge 7mers exhibited strongly-skewed distributions in 22 of these 24 VAEs (stars in Fig. 4b indicate *p* < 0.01 by Fisher’s exact test), revealing subtype-specific responses. Of the *Influenza A virus* VAEs, 4 and 7 were assigned to H1N1 and H3N2, respectively, and these exhibited a strong seasonal effect: 3 of 3 subtype-assignable VAEs in interval 1 mapped to H1N1, and 7 of 8 in interval 2 mapped to H3N2. In contrast, all 11 of the subtype-assignable VAEs for *Enterovirus D* in interval 0 mapped to the D68 subtype.

### Correlation between viral antibody events and orthogonal indicators of infection

The VAEs that we describe here consist of temporally co-ordinated, species-specific patterns of peptide-resolved antibody reactivity that are best explained by contemporary exposures to specific viral antigens, resulting from new infections or reactivations. This interpretation is reinforced by the dynamics of these responses over time (Fig. 2c), as well as the strong epidemic effects that we observe (Figs. 3, 4), attributable to the circulation of viruses in the population. To further test this interpretation, and to chart the evolving antibody responses at finer temporal resolution, we applied PepSeq/PSEA analysis to two additional longitudinal cohorts containing more frequent and continuous longitudinal sampling than the ACS cohort, and also capturing orthogonal time-resolved information about infection events. In both cohorts, high-frequency tracking was enabled using self-collected dried blood spot (DBS) samples and electronic symptom logging (see Methods). To assay this new sample type, we developed an adapted protocol (see Methods), and used it to compare DBS and plasma samples collected at the same time from the same participants. These analyses revealed a highly-significant correlation between the PepSeq assay data on the different sample types, but with a global signal reduction in DBS relative to plasma (most evident at the lower end of the Z-score range) (Supplementary Fig. 2).

**Fig. 5:**
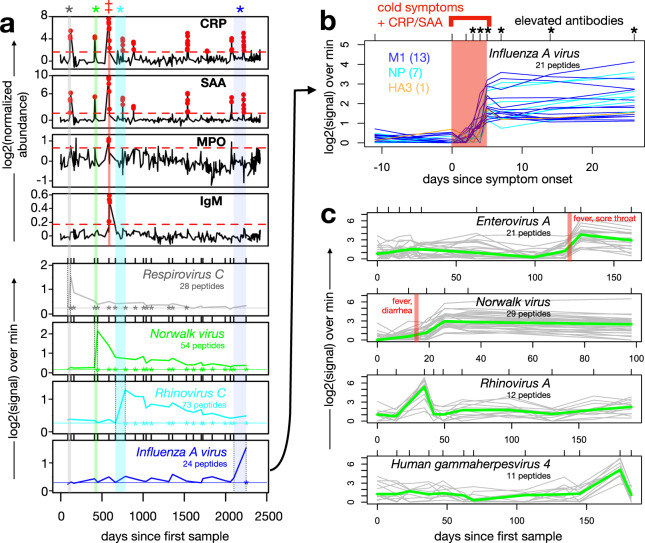
Correlated dynamics of inflammatory proteins, symptoms and anti-viral antibodies measured in longitudinal dried blood spot sample series. **a** 379 serial dried blood spot samples collected from a participant over a ~7 year span were evaluated for abundance of 4 inflammatory proteins—C-reactive protein (CRP), Serum Amyloid A (SAA), Myeloperoxidase (MPO), and total Immunoglobulin M (IgM)—using the SISCAPA assay (upper four panels). Cases where measurements exceeded a threshold (dashed red horizontal lines) at ≥2 consecutive timepoints are highlighted (red dots). A subset of 23 samples across the timespan were also evaluated for anti-viral antibody abundance using PepSeq (lower four panels) in which PSEA detected four significant VAEs (asterisks and vertical shading). The red ‡ indicates the timing of an antibiotic-responsive pneumonia. For each VAE, medians of the leading-edge peptide Z-scores are plotted across all 23 timepoints. Horizontal lines show the median Z-scores of leading-edge peptides at the timepoint immediately prior to the corresponding VAE, and the overlaid asterisks indicate subsequent timepoints whose leading-edge peptide signals are significantly elevated over this baseline (*p* < 0.01 by one-tailed paired *t* test). **b** For the VAE corresponding to Influenza A virus [shown in (**a**)], PepSeq assays were conducted on samples from additional timepoints to fine-map the association between symptoms, inflammatory proteins and virus-specific antibodies. Shown are the PepSeq signals for leading-edge peptides (colored by their protein of origin) across the time series, relative to inflammatory marker elevations and symptoms (upper red bar). Timepoints with significantly elevated antibodies are marked (* indicates *p* < 0.05 by one-tailed t-test). **c** Examples of antibody dynamics detected from serial dried blood spot samples in the ‘MyImmunity’ cohort. Each plot shows the signal across time for VAE leading-edge peptides (gray lines, medians in green) for a species of interest in a different participant. The upper two plots show cases where a VAE coincided with a febrile episode (vertical red bars). The lower two plots show VAEs characterized by rapidly-declining reactivity that returned to baseline within ≤10 days. Tick marks at the top of each plot indicate sampled timepoints. Source data are provided as a Source Data file.

The ‘SISCAPA cohort’ has been described^28^ and comprises volunteers from North America who collected blood spot samples with symptom logs over periods spanning 3-8 years, at variable frequencies up to daily during symptomatic periods. We focused our analysis here on a single healthy participant (“S-18”) with the longest series: 379 samples, collected over a span of ~7 years (Supplementary Fig. 4b and Supplementary Data 5). Inflammatory markers were quantified from the DBS samples using the previously-described SISCAPA assay in which monoclonal antibodies are used to enrich defined peptide fragments of analytes of interest from trypsinized samples, prior to peptide quantification by Mass Spectrometry and normalization against isotopically-labeled control peptides^29^. This analysis revealed a total of 9 ‘inflammatory events’ involving C-reactive protein (CRP) ± Serum Amyloid A (SAA), Myeloperoxidase (MPO) and/or total Immunoglobulin M (IgM), which we define here as protein elevations above a threshold for ≥2 consecutive timepoints (Fig. 5a, red dots in upper 4 panels).

We next selected 23 of the 379 samples, distributed across the time-series, for antibody analysis by PepSeq (Fig. 5a, lower 4 panels). We applied PSEA to the resulting data and identified 4 strong VAEs corresponding to human *Respirovirus C*, *Norwalk virus*, *Enterovirus C* and *Influenza A virus* (Fig. 5a, vertical shaded regions). These VAEs overlapped 4 of the 9 inflammatory events, and no VAEs were detected in the 13 antibody sampling windows that lacked an inflammatory event, revealing a significant overall correlation between antibody and inflammatory events (*p* = 0.017 by Fisher’s exact test). The leading-edges for the 4 VAEs comprised 24-73 peptides, whose kinetics uniformly consisted of a rapid increase during the sampling window that overlapped the inflammatory event, followed by a slower but progressive decline. For the 3 VAEs with >1 month of post-event sampling, antibody responses against leading-edge peptides remained significantly elevated for years after the event (~4 years, >5 years, >4 years for *Respirovirus C*, *Norwalk virus*, *Rhinovirus C*, respectively). For the *Influenza A virus* event, which was also accompanied by the symptoms of a cold, we fine-mapped the temporal association between the antibody and inflammatory signals by performing PepSeq analysis on additional samples collected at days −11, 0, 2, 3, 4, 5, 7, 14 and 27 relative to the onset of the inflammatory event (Fig. 5b). Compared to the pre-event baseline, antibodies became significantly elevated beginning on day 3 and increased sharply until day 5, with continued increases up to day 27 but at a slower rate. The response was dominated by reactivities against the conserved M1 and NP proteins, and is consistent with the kinetics of a recall antibody response, likely primed by previous Influenza A vaccinations/infections.

The ‘MyImmunity’ cohort comprises 30 healthy adult volunteers residing in Arizona who each collected 5–20 (median 10 per participant, total 358) DBS samples and associated symptom logs each on a weekly-monthly basis during June–December 2019 (Supplementary Fig. 4c and Supplementary Data 6). In total, 4 participants reported febrile episodes, 2 of which coincided with VAEs, corresponding to *Enterovirus A* and *Norwalk virus*, respectively (Fig. 5c, upper two rows). In addition to fever, each episode included classic symptoms of each virus: sore throat for *Enterovirus A* and diarrhea for *Norwalk virus*. In this frequently-sampled cohort, we also observed examples of a unique type of VAE not seen in the previous cohorts: events with rapidly-declining kinetics in which reactivity returned to baseline within periods of 1–3 weeks following the VAE (Fig. 5c, lower two rows). These events were restricted to Rhinovirus species and EBV, representing viruses against which we detected some of the highest levels of overall baseline reactivity. Such kinetics may result from abortive infections/reactivations that cause transient waves of plasmablast activity recruited from the memory B cell pool, but without triggering extensive new affinity maturation or formation of new long-lived plasma cells.

## Discussion

In this study, we have developed an approach for the virome-wide detection of infection events within longitudinal high-dimensional antibody measurements and used this system to study viral and host dynamics in individuals and populations over timescales ranging from days to years. Our approach combines a highly-multiplexed serological assay platform with a repurposed statistical tool for the analysis of the resulting time-resolved, high-dimensional reactivity data (Fig. 1). In our analysis of longitudinally-sampled cohorts, we identified 100s of species-specific ‘viral antibody events’ (VAEs) that consist of temporally-co-ordinated changes in reactivity to epitopes across the respective proteomes (Fig. 2). We ascribe these serological events to co-incident viral infections or reactivations, an interpretation supported by the temporal association between VAEs and orthogonal markers of infection (Fig. 5), as well as the strong epidemic effects that we observe in a synchronously-sampled cohort (Fig. 3).

Like all serological assays for infection, our approach is limited by the kinetics of the antibody response, and so becomes sensitive later during the course of infection than direct viral detection. Nonetheless, our detection of significant species-specific signal within as few as 3 days after symptom onset (Fig. 5b, c) suggests that longitudinal sampling in the early symptomatic period may be diagnostic in the setting of a recall response, which likely applies to the majority of natural human infection events. Nonetheless, the advantages of the longitudinal, highly-multiplexed approach are likely strongest in the context of population-level surveillance or retrospective correlative studies that do not require the timely detection of individual cases. While low-dimensional serology has also been used effectively for such purposes, the highly-multiplexed peptide-based approach offers a number of important advantages, including: (i) an ability to simultaneously query many infectious agents, (ii) increased statistical power resulting from the detection of correlated signals across many proteins/epitopes per species target, (iii) increased taxonomic discrimination resulting from epitope-level resolution, and (iv) the generation of rich background distributions that allow more accurate normalization across samples with global differences in signal quality. The latter consideration may be particularly important for cohorts that would otherwise be confounded by sample-to-sample variability due to differences in storage/degradation (e.g. dried blood samples), which may pose particular challenges in longitudinal studies.

While the statistical approach that we describe allows viral events to be inferred with high-confidence and specificity, the natural history cohorts studied here do not allow a precise quantification of the sensitivity with which it detects true infection events. There are, however, several observations that suggest a relatively low false negative rate. First, the overall frequency of events detected in the ACS cohort (~5 per person-year, ~50% of which correspond to respiratory viruses), is broadly consistent with published estimates^3,30,31^. Second, the fractions of inflammatory or febrile episodes in the SISCAPA and MyImmunity cohorts for which we detect a VAE—4/9 and 2/4 respectively—is relatively high, particularly in light of the potential for such episodes to have non-viral causes. Third, we detect epidemic waves affecting large subsets (5-30%) of the cohort, including one for *Influenza A virus* encompassing ~20% of participants, a value that is consistent with estimates for the annual incidence in the United States of this well-studied virus^32,33^. Future studies in which the approach described here is paired with the direct detection of viral nucleic acids or proteins will be useful to compare the sensitivities of these approaches, although such studies will need to be designed to account for the transient and often site-specific appearance of viral components during an infection.

The observation that all four VAEs that we detected in the SISCAPA cohort are associated with elevations of both CRP and SAA (Fig. 5) suggests that time-dense measurements of these inflammatory markers can provide a sensitive measure of viral infection. It is notable that the strongest inflammatory event detected here (which also includes elevations in MPO and IgM; marked by red vertical stripe in Fig. 5a) did not associate with an inferred viral event, but instead coincides with an episode of X-ray-confirmed antibiotic-responsive pneumonia. We hypothesize that some or all of the four other inflammatory events that lack a co-incident VAE could likewise be explained by infections with non-viral agents, but without being severe enough to reach clinical attention and involve MPO/IgM elevations. Alternatively, a subset of these events may be attributable to viral infections to which our approach is insensitive, potentially related to the signal loss we observed in DBS samples (Supplementary Fig. 2). These hypotheses should be testable in future studies that apply highly-multiplexed serology, such as PepSeq, to measure antibody reactivity to bacterial and/or fungal antigens in longitudinal series of this type. Future studies in larger cohorts will also be important to determine how the overall association between viral antibody and inflammatory profiles described in Fig. 5 generalizes beyond the single participant shown here.

The potential of our approach to yield valuable epidemiological insights is exemplified by the detection of high-prevalence epidemic waves for *Aichivirus A* and the D68 subtype of *Enterovirus D*in the ACS cohort (Fig. 3). The observation of 11 synchronous Enterovirus D68 events in the Western Cape province of South Africa during 2006 is striking and would represent the largest known cluster of infections of its time for this virus^34^. Enterovirus D68 came to prominence in the autumn of 2014 when it was associated with a large outbreak of pediatric lower-respiratory disease in North America and Europe, along with >100 cases of acute flaccid paralysis in the United States^35^. It has since caused smaller, biennial outbreaks in the summer-to-autumn season. Although the peak of the wave in our cohort appears to have preceded our sampling window (which began mid-2006), extrapolation from the timing of decreasing reactivity (Fig. 3b) suggests a peak of infections ~April of 2006, coinciding with the Southern Hemisphere autumn season. Our observation of a high-incidence (20%) outbreak in this adolescent surveillance cohort that was unselected for active respiratory disease or symptoms is consistent with the model that Enterovirus D68 is a widely-circulating virus that only comes to clinical attention in a small minority of cases^36^. Significant circulation in South Africa in 2006, despite the absence of a recognized major clinical outbreak until 2014, might be explained by geographically-related differences in host susceptibility, evolution of the virus during the intervening years, and/or limitations in the available surveillance tools. Similarly, our detection of a wave of *Aichivirus A* infections affecting ~30% of participants over the Southern Hemisphere summer of 2007-2008 (Fig. 3b) to our knowledge represents the highest incidence wave ever detected for this virus in a general cohort and the earliest documented evidence of its widespread circulation on the African continent^37,38^.

Also notable is our detection of (non-epidemically-clustered) VAEs across 7 members of the Human Herpesvirus (HHV) family (Fig. 2a). The HHVs establish life-long infections characterized by long periods of latency, but are known to sometimes reactivate, typically in conditions of stress or immunosuppression, and sometimes with important clinical consequences^39^. The fact that we detect pre-existing reactivity prior to each HHV event indicates that these VAEs are best explained as viral reactivations, as opposed to primary infections, although re-infections are also possible. Moreover, although the ACS cohort lacks the clinical records necessary to track individual outcomes, the frequencies of these events (e.g. 28 and 11 for *Human betaherpesvirus 5* and *Human alphaherpesvirus 3*, respectively) far exceed the expected incidences of their respective diseases (CMV reactivation disease and Herpes Zoster), indicating that most were subclinical or asymptomatic^40,41^. The possibility that serological profiling of frequent, subclinical HHV reactivations could serve as a sensitive and dynamic reporter of a person’s immunological health is an intriguing area for future study. Also remarkable is the detection of VAEs for *Measles morbillivirus* and *Rubivirus rubellae* in the ACS cohort, representing species whose circulation is increasingly curtailed by highly-effective childhood vaccination. The detection of 8 Rubella events (1 per ~15 person-years) is particularly notable as it reveals significant circulation in this population, likely reflecting the absence of Rubella in the standard childhood vaccination schedule in South Africa^42^.

We expect the approach developed here to find application in future studies of epidemiological patterns in cohorts sampled longitudinally over timescales of months-years, or more fine-level immune dynamics in individuals sampled over days-weeks. Our adaptation of the PepSeq assay to self-collected dried blood spot samples (Fig. 5) will also enable new, scalable study designs with temporal resolution not readily achieved with traditional clinical collections. Moreover, since highly-multiplexed peptide-based serology platforms like PepSeq are fully-customizable in their antigen content, it should be possible to directly extend the experimental and analytic approaches described here to other areas of epidemiological or clinical interest: for example to enable the broad identification of bacterial infection or allergen exposure events.

## Methods

### Cohorts

The COVID-19 vaccine cohort has been previously described^15^ and comprises 21 participants aged 18–60+ with no known history of SARS-CoV-2 exposure who received 2 doses of the mRNA-1273 vaccine and from whom plasma was collected at days 0, 8, 28 and ~140 following the first dose. The Adolescent Cohort Study (ACS) was conducted during 2006–2008 and consists of 12–18 year-old *Mycobacterium Tuberculosis*-infected participants residing in the Western Cape of South Africa, as described previously^22^. We focused here on a subset of 260 samples from 65 participants with evidence of *Mycobacterium tuberculosis* infection based on a positive tuberculin skin test or interferon-gamma release assay^43^, for whom the complete time-series of 4 × 6-monthly samples were available. Blood samples were processed to generate plasma and stored at −20 °C prior to their evaluation by PepSeq. The ‘SISCAPA cohort’ has also been described^28^ and comprises volunteer participants from North America who collected blood spot samples with symptom logs over periods spanning 3-8 years, at variable frequencies up to daily during symptomatic periods. We focused our analysis here on a single 60+ year-old healthy male participant (“S-18”) with the longest series: 379 samples, collected over a span of ~7 years. The ‘MyImmunity cohort’ comprises 30 healthy adult volunteers aged 18–58+ residing in Arizona who each collected 5–20 (median 10 per participant, total 358) dried blood samples and associated symptom logs each on a weekly-monthly basis during June–December 2019. The COVID-19 vaccine and MyImmunity cohorts were collected under IRB-approved protocols (Western IRB #20191236). The ACS study was approved by the Faculty of Health Sciences, Human Research Ethics Committee of the University of Cape Town. The SISCAPA study was approved by Advarra under IRB#00000971. All cohorts gave written informed consent: in the case of the ACS study, written informed consent was obtained from the parents of the adolescents and assent was obtained from the adolescents.

### Dried blood spot collection and processing

For the SISCAPA cohort, dried blood spots were collected on Whatman 903 Protein Saver cards and stored at 4 °C (for 4–10 years depending on the collection date) under low humidity conditions. For the MyImmunity cohort, dried blood samples were collected onto Whatman 903 Protein Saver cards and stored under ambient conditions for 0–80 (mean ~20) days prior to reconstitution. To generate input material for PepSeq assays, 6 mm punches were collected from the cards and antibodies were resolubilized in 100 µL of PBS at room temperature for 1 h. After removal of the filter paper, reconstituted blood spot solution was stored at −20 °C prior to assays.

### PepSeq assays

Highly-multiplexed, epitope-resolved IgG reactivity analysis across the human virome was performed on plasma (ACS cohort) or reconstituted dried blood (SISCAPA and MyImmunity cohorts) using DNA-barcoded peptide (“PepSeq”) assays. The COVID-19 vaccination cohort was assayed using the ‘HV2’ library, which has been previously described^15^ and consists of 15,000 30mer peptides covering 80 viral species and selected based on prior evidence of reactivity in other cohorts (sequences available in Supplementary Data). The ACS and SISCAPA cohorts were assayed using the ‘HV2T’ library, which consists of 15,000 64mer peptides covering the same 80 viral species (see Supplementary Data 1). The HV2T peptide content overlaps with that of HV2 but using 64mer sequences configured as tandem 30mers, each separated by a 4-amino acid spacer (“SGSG”). Samples from the MyImmunity cohort were assayed using a 244,000-member 30mer human virome-wide library (‘HV1’; the precursor to HV2 and HV2T) designed to cover maximum 9mer amino acid sequence diversity across the proteins of all viruses annotated to have human tropism, as previously described^14^.

PepSeq libraries were synthesized and used to profile IgG binding as previously described^14,16^. Briefly, DNA-barcoded peptide libraries were generated using bulk in vitro enzymatic reactions, starting with the PCR amplification of oligonucleotide templates and their transcription to generate mRNA. The product was ligated to a hairpin oligonucleotide adaptor bearing a puromycin molecule tethered by a PEG spacer and used as a template in an in vitro translation reaction. Finally, a reverse transcription reaction, primed by the adaptor hairpin, was used to generate cDNA, and the original mRNA was removed using RNAse. To perform serological assays, 0.1 pmol of the resulting DNA-barcoded peptide library (5 uL) was added to 5 uL of sample (either plasma diluted 1/10, or neat reconstituted blood spot solution) and incubated overnight. The binding reaction was applied to pre-washed protein G-bearing beads, washed, eluted, and indexed using barcoded DNA oligos. Following PCR cleanup, products were pooled, quantified and sequenced using an Illumina NextSeq instrument.

### Longitudinal data analysis

Z-score enrichment signals for each sample-peptide combination were generated from raw sequence reads in a 2-step process using *PepSIRF* v1.4.0, an open-source software package that we previously developed for this purpose^16,44^. First, reads were demultiplexed and mapped to members of the respective HV2T, HV2 and HV1 libraries using the *demux* module to generate integer counts values for each sample-peptide. Next, peptides with similar abundances in the buffer-only negative control samples were grouped into bins and used to generate Z-scores for each datapoint, representing the distance (in standard deviations) of each datapoint from its unenriched distribution mean. In addition to buffer-only negative controls, a positive control plasma sample with known reactivity status across a panel of viruses was included on each assay plate and used to ensure plate-to-plate consistency (Pearson’s R > 0.95) in signal (Supplementary Fig. 2). Samples were also clustered by pairwise similarity of their Z-scores (using the *cor* function in R) to verify the expected co-clustering of samples by participant identity and to exclude any non-clustering outliers (<1% of samples, interpreted as mix-up/contamination events). Log2-transformed offset-adjusted Z-scores (log2(Z + 8) − 3) (hereafter, ‘transformed Z-scores’) from all remaining samples were used for all downstream analyses. To identify species-specific temporal changes, we implemented Peptide Set Enrichment Analysis (PSEA) using the *GSEA* function in the R *clusterProfiler* library v4.2.2^45^—with peptides in the place of genes, and species in the place of pathways/functional groups. We first used the *smooth.spline* R function to fit a cubic spline to the transformed Z-scores across the data from each focal pair of timepoints (‘interval’). For each peptide, the residual between the observed transformed Z-score at the later timepoint and its spline-predicted value across the pair—reflecting the magnitude and direction of its temporal change across the interval—was used as input for the *GSEA* function. Residuals for all peptides above a transformed Z-score threshold of 0.75 at either timepoint were associated with their respective viral species of design and passed into *GSEA* to calculate adjusted *p* values, Normalized Enrichment Scores and leading-edge subsets for each of species for which at least 3 peptides passed our thresholds. Species-interval combinations with *p* values < 0.01 (adjusted by the Benjamini & Hochberg method), and for which the strongest peptide signal at either timepoint had a transformed Z-score ≥1.5, were classified as VAEs. These methods were applied uniformly (i.e., with common thresholds) across all species.

To analyze peptide dynamics across >2 timepoints, leading-edge peptides identified for a species of interest across a focal pair of samples were displayed (as individual spline-normalized Z-score traces, or timepoint-wise medians) or compared to each other (using Student’s t-test) following spline adjustment. For this purpose, the timepoint-wise spline normalization approach described above was generalized to >2 timepoints by fitting splines between each timepoint and the peptide-wise median signals across all timepoints in the series, and taking residuals.

For seasonality analysis, we used the *rmultinom* function from the stats package (4.2.2) in R to perform random trials of a multinomial model in which the total number of VAEs observed for each species of interest was distributed across 4 intervals under null conditions of uniform probability. *P* values were calculated as the frequency of such trials in which the number of events assigned to the season with the most events met or exceeded the corresponding observed value for that virus.

For sub-species analysis of *Influenza A virus* and *Enterovirus D*, we performed exact match alignment of all possible 7mers in the leading-edge peptides for each VAE against the following reference protein sequences (UniProt accessions): Influenza A H1N1 (Q6WG00, Q7TG96, Q1K9M9, Q1K9N2, Q1K9M7), Influenza A H3N2 (W0RXT2, B4URC8, Q0PEM7, C9S3S7, B2BUJ3), Enterovirus D68 (Q68T42), Enterovirus D70 (P32537), Enterovirus D94 (ABL61316.1).

### ELISA validation

ELISA kits for anti-Influenza A virus IgG (Alpha Diagnostic International, cat# 920-040-HAG) and anti-CMV IgG (Calbiotech, cat #CM027G) were run according to the manufacturers’ instructions, unless otherwise specified. Samples from timepoint pairs that either spanned or did not span a PepSeq VAE were assayed in duplicate (i.e., 4 assays per interval) at fixed sample dilutions determined to be optimal in preliminary assays: 1:297 for Influenza virus and 1:63 dilution for CMV (in each case, one-third of the manufacturer’s recommended dilution). Duplicates were averaged and the difference in raw optical density values between consecutive timepoints was taken and plotted according to PepSeq VAE status.

### SISCAPA assays

For this study we used the ‘acute inflammatory response panel’ to quantify the following proteins in longitudinal DBS samples: SAA, CRP, LPSBP, Hp, Hx, FibG, MBL, A1AG, C3. Briefly, The DBS samples were digested using trypsin as a protease before monoclonal antibody reagents (SISCAPA Assay Technologies) were used to enrich proteotypic peptides unique to the proteins of interest from the digested matrix. Stable isotope labeled internal standard peptides, corresponding to each of the endogenous peptides being measured, were spiked into the sample at known concentrations. The ratio of the endogenous peptide to the internal standard was used to quantitate the endogenous levels of the respective analyte. The identification and quantitation of the peptides was performed using an LC-MS/MS configuration consisting of an Eksigent nanoLC 425 system operating at 10 µL/min coupled to a QTRAP 6500 mass spectrometer (Sciex, USA). The target peptides were separated using a 10-min analytical gradient with 0.1% formic acid (FA)/5% DMSO in water as solvent A and 90% acetonitrile/5% DMSO in 0.1% FA in water as solvent B. The peak area ratios were analyzed using MultiQuantTM software (SCIEX).

### Statistics and reproducibility

Viral Antibody Events were identified using Peptide Set Enrichment Analysis, as described above (Longitudinal data analysis, Methods section), at a threshold of adjusted *p* value < 0.01. For other comparisons, we applied t-tests, Fisher’s exact tests, Wilcoxon Rank Sum tests, Pearson’s product-moment correlation tests or binomial/multinomial models, as stated in each of the Results and Figure Legends sections. The renv package (0.16.0) was used for management of the R packages used in this study, and the associated R project and packages are available in the Open Science Framework repository. All viral profiles were generated blinded to the covariates (sex, age, disease status) and then unblinded for covariate analysis. No statistical method was used to predetermine sample size. No data were excluded from the analyses. The experiments were not randomized.

### Reporting summary

Further information on research design is available in the Nature Portfolio Reporting Summary linked to this article.

## Supplementary information

Supplementary Information

Reporting Summary

## Data Availability

All data generated in this study, including both raw and processed matrices, have been deposited in the Open Science Framework database under 10.17605/OSF.IO/6HT43 [https://osf.io/6ht43/]^46^. Source data are provided with this paper.

## Source data

Source Data

## Code availability

All code for this study is available in the Open Science Framework database under 10.17605/OSF.IO/6HT43 [https://osf.io/6ht43/]^46^.
