# Supplementary material for: Virome-wide detection of natural infection events and the associated antibody dynamics using longitudinal highly-multiplexed serology
Source: Nat Commun. 2023 Mar 30;14:1783. doi: 10.1038/s41467-023-37378-z (PMC10062260; doi:10.1038/s41467-023-37378-z)
Supplement: Supplementary file 1 — Supplementary Information [file 41467_2023_37378_MOESM1_ESM.pdf]

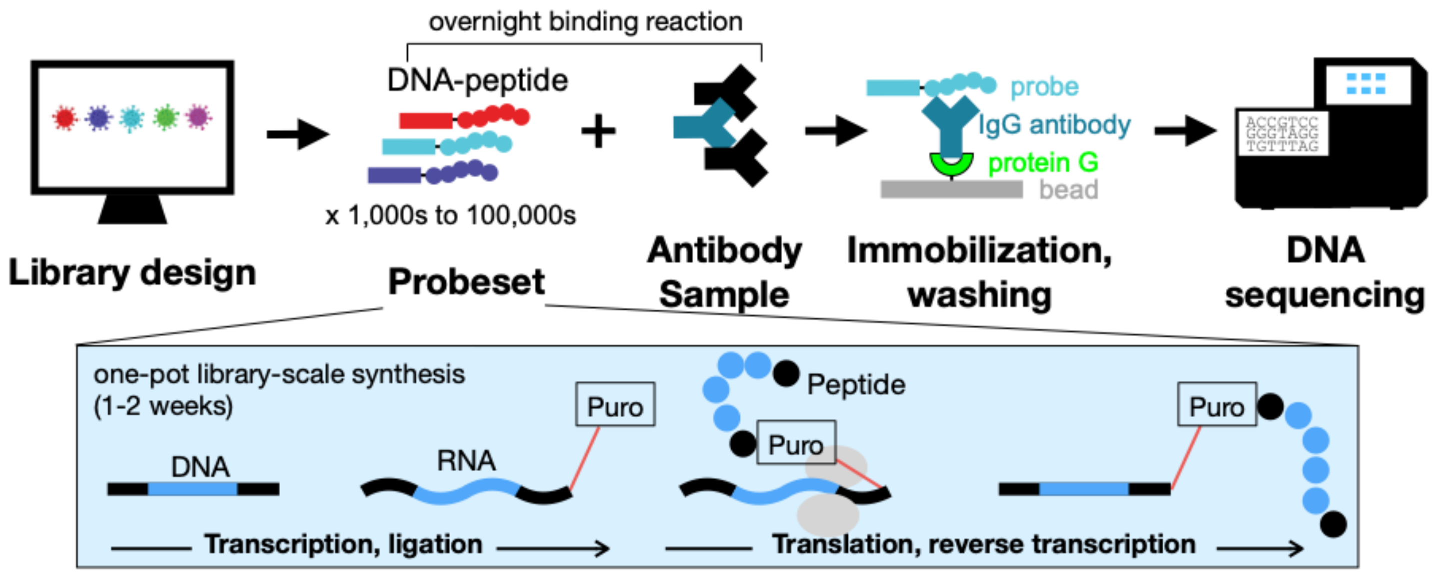

Supplementary Figure 1: Highly-multiplexed antibody analysis at epitope-resolution using PepSeq. Beginning with an in silico-designed library of 1,000s-100,000s of desired peptide sequences (in this case, representing the human virome), PepSeq allows a highly-diverse probset of DNA-barcoded peptides to be synthesized in a single reaction using in vitro transcription and translation followed by covalent intramolecular peptide:mRNA coupling using puromycin ("Puro", see lower panel). Following incubation of this probset with a plasma sample, binding can be quantified across the library by immobilizing the antibodies on beads, washing, and then amplifying and sequencing the DNA tags of bound probes.

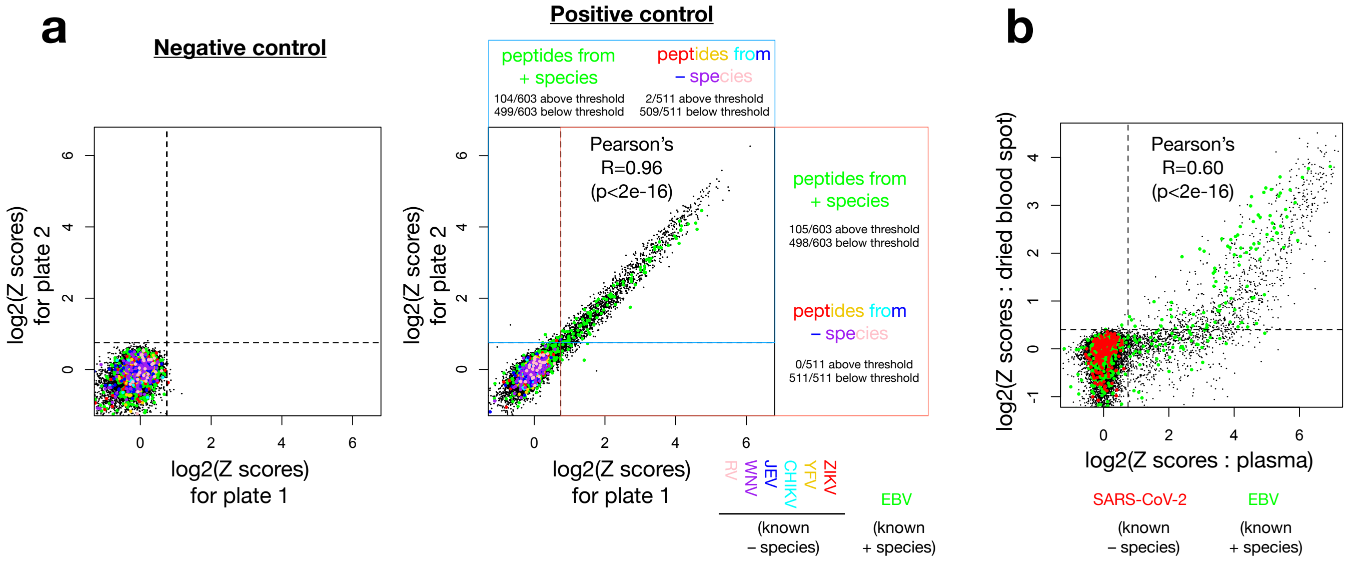

Supplementary Figure 2: Library-wide analysis of control samples showing assay dynamic range, thresholds used, replication across assay plates and correlation between plasma and DBS sample types. **(a)** Each assay plate includes a negative control sample (*left*) containing buffer alone, and a positive control sample (*right*) comprising plasma from a donor with known reactivity to *Human gammaherpesvirus 4* / Epstein Barr Virus (EBV) ("+ species"), but no history of exposure or vaccination to *Zika virus* (ZIKV), *Yellow fever virus* (YFV), *Chikungunya virus* (CHIKV), *Japanese encephalitis virus* (JEV), *West Nile virus* (WNV) or *Rabies lyssavirus* (RV) ("– species"). Shown is assay signal (log2(Z scores): calculated as described in the Methods section) for each of the 15,000 peptides in the HV2T library comparing control sample replicates across two different plates/days (X and Y axes). Each dot represents an individual peptide: those designed from the known positive (n=603) or negative (n=511) species are highlighted in the indicated colors, while peptides from the remaining 73 species are shown as smaller black dots. Dashed horizontal and vertical lines show the thresholds used to select peptides as input for PSEA analysis, and the number of +/– species-derived peptides above these thresholds are indicated for the positive control. **(b)** Representative scatterplot analysis of library-wide peptide signals comparing plasma (x-axis) v Dried Blood Spot (y-axis) samples collected simultaneously from the same donor (different from the positive control donor shown in a), who is known to be negative for *SARS-CoV-2* (peptides in red) and positive for EBV (peptides in green). P values were calculated using Pearson's product-moment correlation.

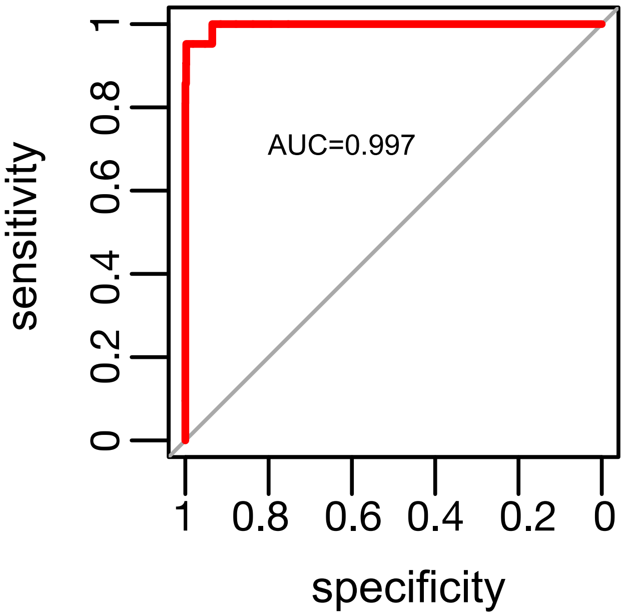

Supplementary Figure 3: Benchmarking of PSEA using a longitudinal COVID-19 vaccine cohort. Samples from 21 participants collected pre- and post-vaccination were assayed in duplicate using the HV2 PepSeq assay and analyzed using PSEA. Shown is Receiver-Operating Characteristic curve analysis of p-values comparing expected positives (signals at day 0 v day 140 for *SARS-CoV-2* in each participant, n=21) and expected negatives (signals in replicate assays at each timepoint across all 80 species in each participant, n=5360).

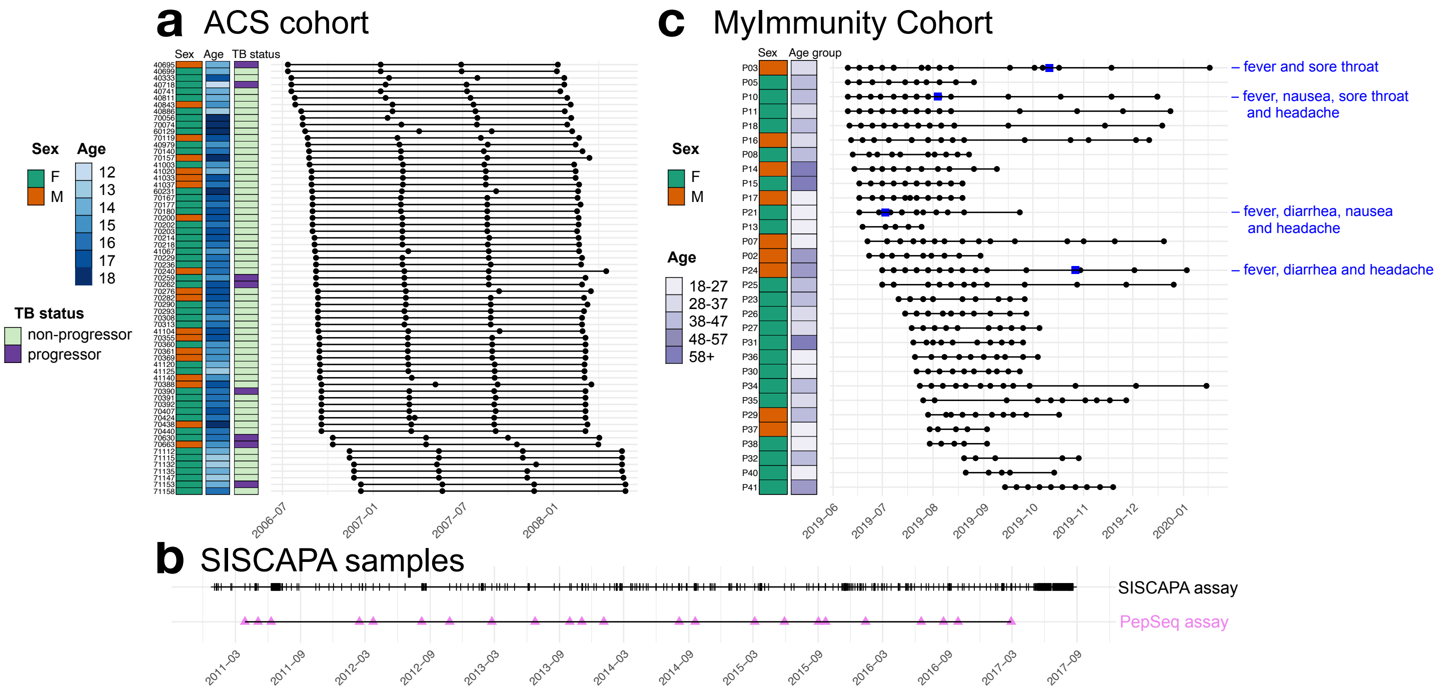

Supplementary Figure 4: Sampling schemes for the three cohorts studied: ACS (a), SISCAPA (b) and MyImmunity (c). Each horizontal line represents a unique participant, and symbols along the line represent sampling timepoints, marked against the timescale on the x-axis. Blue markings in panel **b** indicate the timing of febrile episodes, whose respective symptoms are indicated on the right.

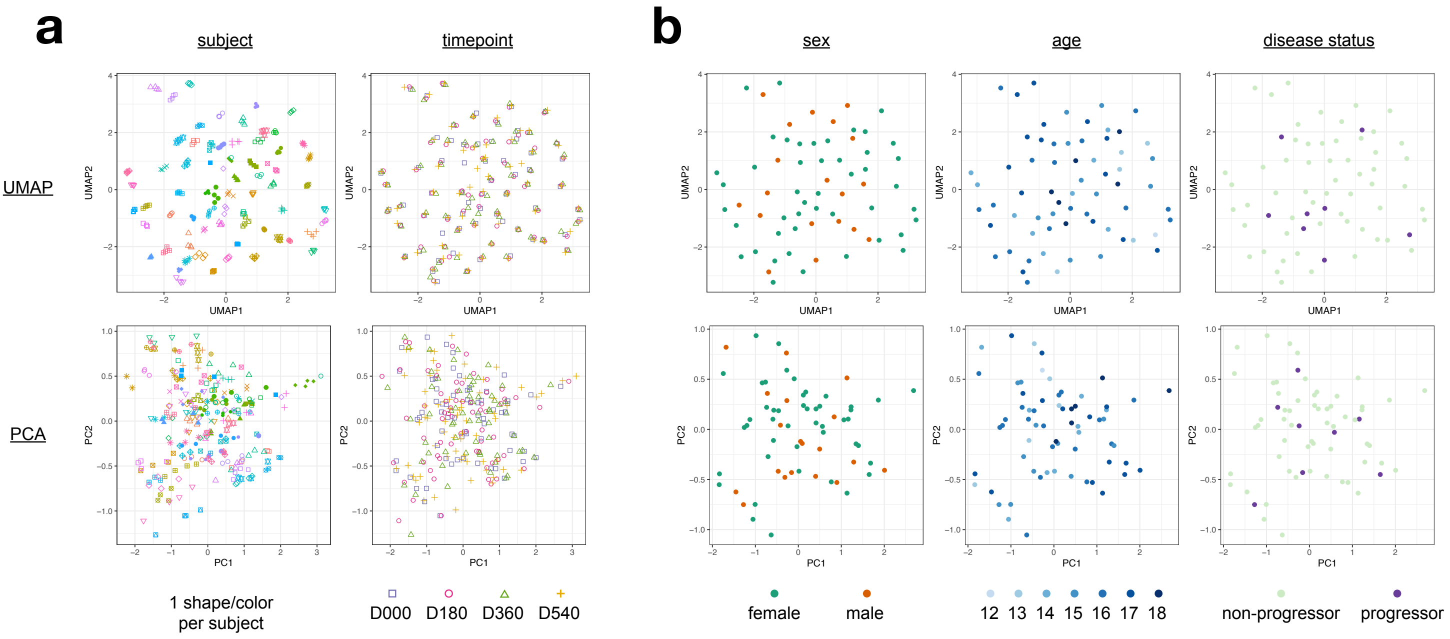

Supplementary Figure 5: Unsupervised clustering reveals donor-specific signatures, but no effect of sex, age or disease status on global patterns of peptide signal. (a) UMAP (*upper*) and PCA (*lower*) clustering of global peptide signals across all samples (65 donors x 4 timepoints = 260 total samples) in the ACS cohort, marked by donor (*left*) or timepoint (*right*). **(b)** UMAP and PCA clustering as in (a) but now showing one sample (timepoint 0) from each donor (65 total samples), and marked by sex (*left*), age (*center*) and disease status (*right*).

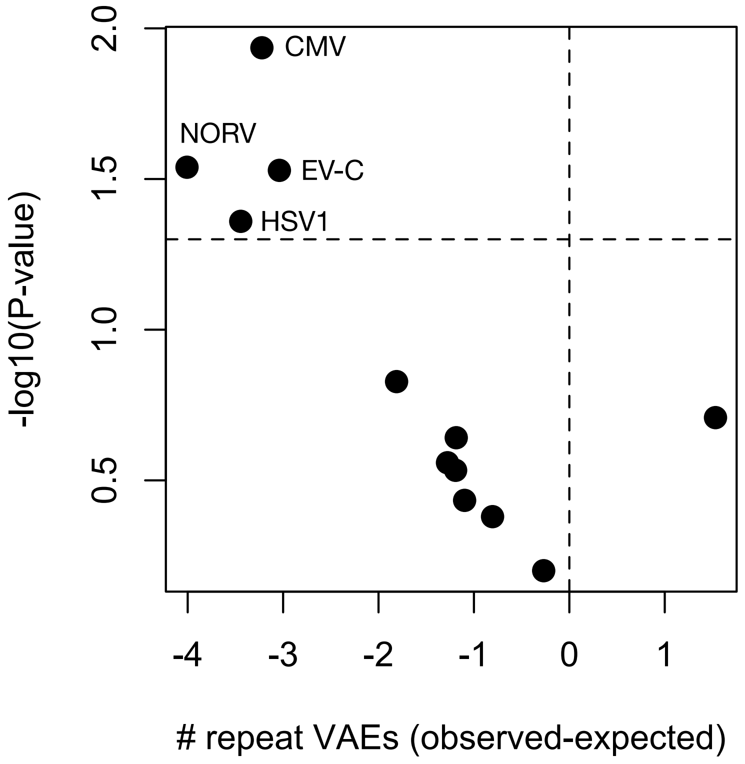

Supplementary Figure 6: Frequency of 'repeat VAEs' for each species compared to a null model. To test whether multiple VAEs from the same viral species in the same participant (repeat VAEs = 'rVAEs') occur more/less frequently than expected, we generated a one-sided multinomial model in which the events observed in the ACS cohort (matched by interval) were randomly permuted across the 65 participants. We generated p-values (y-axis) by quantifying the fraction of such permutations (among 10,000 generated for each species) that yielded a rVAE count that was equal to or more extreme than the count observed in the original (unpermuted) data. To maximize statistical power, the analysis was restricted to the 12 viral species for which ≥20 total VAEs were observed across the cohort, each shown as a single dot. Species with unadjusted $p<0.05$ (threshold indicated by horizontal dashed line) are labeled as follows: CMV = *Human betaherpesvirus 5*; NORV = *Norwalk virus*; EV-C = *Enterovirus C*; HSV1 = *Human alphaherpesvirus 1*.

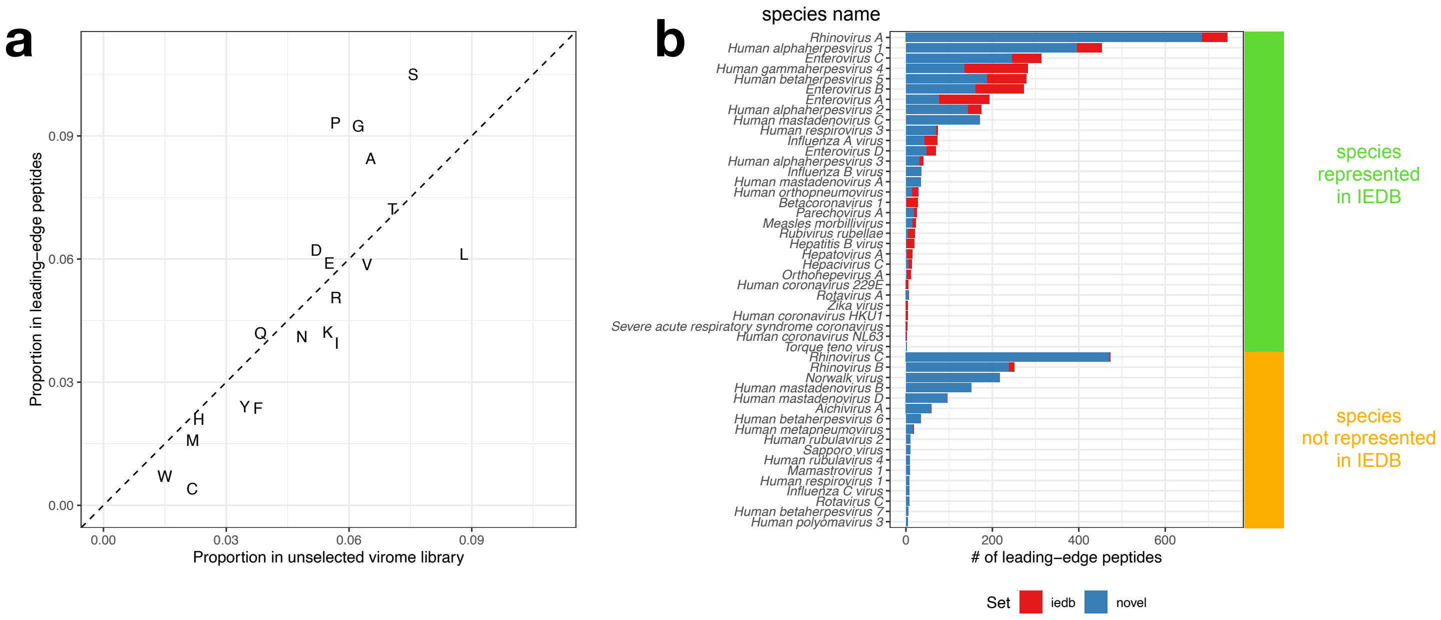

Supplementary Figure 7: Analysis of amino acid composition and epitope sequence content of peptides recognized by responding antibodies. Leading-edge peptides from all significant VAEs detected in the ACS cohort were combined and analyzed for their **(a)** amino acid composition and **(b)** degree of overlap with human antibody epitopes reported in the Immune Epitope Database (IEDB), which compiles epitopes from the literature. In **a**, the aggregate frequency of each amino acid (represented by its single letter code) in the leading edge peptides (y-axis) is shown as a function of its frequency in the unselected virome-wide library HV1 (x-axis, representing the virome amino acid frequency baseline). In **b**, leading-edge peptides sharing ≥1 9mer with any linear epitope to which a positive human antibody response was reported in IEDB (downloaded on September 7, 2022) are considered an IEDB epitope match (red); remaining leading-edge peptides are considered novel (blue). Peptides are organized by species and displayed according to whether each species either has (*upper, green*) or lacks (*lower, orange*) representatives in IEDB. Matches to IEDB in the lower section (eg red bar in *Rhinovirus B*) result from sequence conservation with a species present in IEDB.

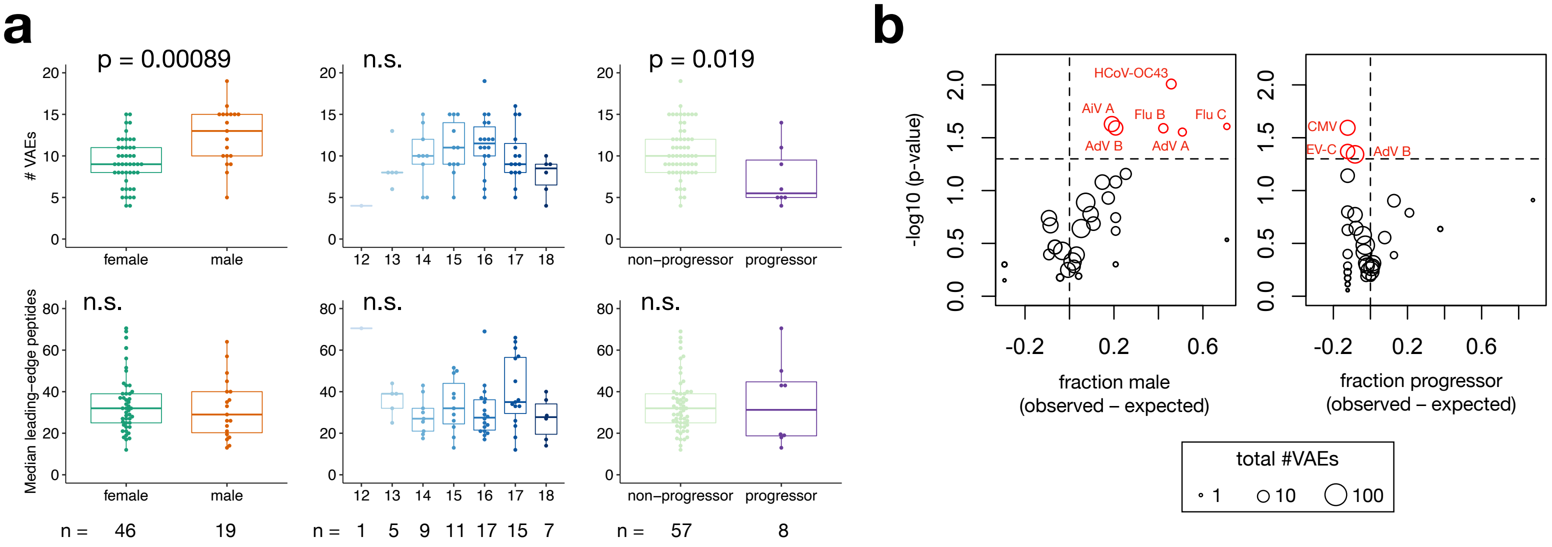

Supplementary Figure 8: Influence of sex, age and disease status on the number of Viral Antibody Events (VAEs) and Leading Edge (LE) peptides detected. (a) Boxplots showing the number of VAEs (*upper*) and median leading-edge peptides per VAE (*lower*) for each participant in the ACS cohort, organized by sex (*left*), age (*center*) and disease status (*right*). Numbers of participants in each group are indicated below. Significance was tested using two-sided Wilcoxon Rank Sum tests (in the case of age, all pairwise comparisons were performed and then adjusted for multiple comparisons) and comparisons with $p<0.05$ are indicated. The bottom and top of the boxes mark the first and third quartiles (respectively), the center line in the boxes marks the median, and the whiskers extend from the boxes to points within 1.5 times the interquartile range of the box limits. **(b)** Volcano plots quantifying the degree of gender (*left*) or disease status (*right*) imbalance in the observed VAE distributions for each viral species with ≥1 VAE in the ACS cohort. Unadjusted p-values (y-axis) were calculated using a one-sided binomial model in which the observed count of VAEs for each species was re-distributed in a covariate-independent way in 1,000,000 random trials, and the direction of the effect (x-axis) was calculated by normalizing the observed covariate distribution for each virus against the distribution of the overall cohort. Species above the thresholds shown are marked in red and labeled as follows: AdV A = *Human mastadenovirus A*; AdV B = *Human mastadenovirus B*; HCoV-OC43 = *Betacoronavirus 1*; Flu B = *Influenza B virus*; FluC = *Influenza C virus*; AiV A = *Aichivirus A*; CMV = *Human betaherpesvirus 5*; EV-C = *Enterovirus C*.

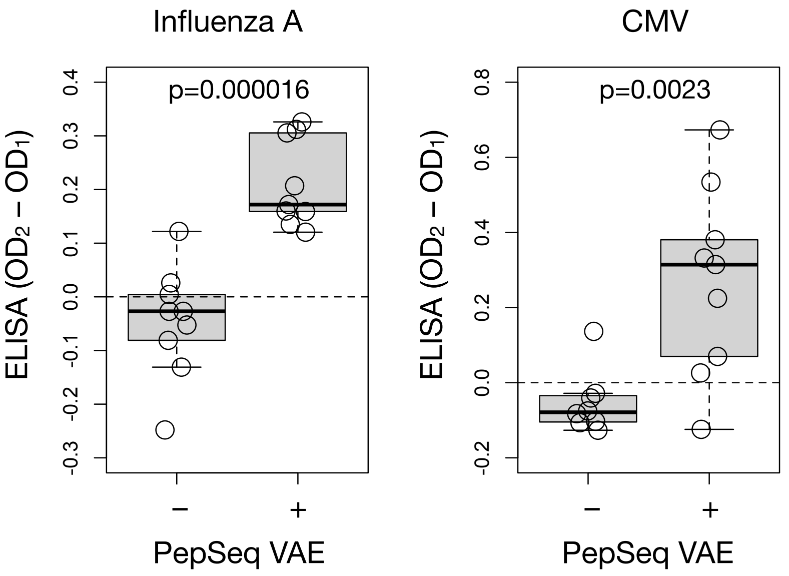

Supplementary Figure 9: Comparison of signal from PepSeq v ELISAs across consecutive longitudinal samples. Sample pairs spanning intervals that either contained or lacked a PepSeq VAE (x-axis groups; determined using the analysis shown in Figure 2) for *Influenza A virus* (*left*) or *Human betaherpesvirus 5* / CMV (*right*) were assayed in duplicate by commercial ELISAs that detect IgG reactivity against the respective native viruses. The difference in raw Optical Density between the timepoints for each donor is shown on the y-axis, and was compared between PepSeq VAE positive v negative groups using a one-tailed t-test. The bottom and top of the boxes mark the first and third quartiles (respectively), the center line in the boxes marks the median, and the whiskers extend from the boxes to points within 1.5 times the interquartile range of the box limits. From left to right, groups contain n=9, n=9, n=8, n=9 independent participants, respectively.
