# Supplementary material for: Virome-wide detection of natural infection events and the associated antibody dynamics using longitudinal highly-multiplexed serology
Source: Nat Commun. 2023 Mar 30;14:1783. doi: 10.1038/s41467-023-37378-z (PMC10062260; doi:10.1038/s41467-023-37378-z)
Supplement: Supplementary file 2 — Reporting Summary [file 41467_2023_37378_MOESM2_ESM.pdf]

nature portfolio

Corresponding author(s): John Altin

Last updated by author(s): Feb 21, 2023

Reporting Summary

Nature Portfolio wishes to improve the reproducibility of the work that we publish. This form provides structure for consistency and transparency in reporting. For further information on Nature Portfolio policies, see our Editorial Policies and the Editorial Policy Checklist.

Statistics

For all statistical analyses, confirm that the following items are present in the figure legend, table legend, main text, or Methods section.

n/a	Confirmed	
[ ]	[x]	The exact sample size (*n*) for each experimental group/condition, given as a discrete number and unit of measurement
[ ]	[x]	A statement on whether measurements were taken from distinct samples or whether the same sample was measured repeatedly
[ ]	[x]	The statistical test(s) used AND whether they are one- or two-sided<br>*Only common tests should be described solely by name; describe more complex techniques in the Methods section.*
[ ]	[x]	A description of all covariates tested
[ ]	[x]	A description of any assumptions or corrections, such as tests of normality and adjustment for multiple comparisons
[ ]	[x]	A full description of the statistical parameters including central tendency (e.g. means) or other basic estimates (e.g. regression coefficient) AND variation (e.g. standard deviation) or associated estimates of uncertainty (e.g. confidence intervals)
[ ]	[x]	For null hypothesis testing, the test statistic (e.g. *F*, *t*, *r*) with confidence intervals, effect sizes, degrees of freedom and *P* value noted<br>*Give P values as exact values whenever suitable.*
[x]	[ ]	For Bayesian analysis, information on the choice of priors and Markov chain Monte Carlo settings
[x]	[ ]	For hierarchical and complex designs, identification of the appropriate level for tests and full reporting of outcomes
[ ]	[x]	Estimates of effect sizes (e.g. Cohen's *d*, Pearson's *r*), indicating how they were calculated

Our web collection on statistics for biologists contains articles on many of the points above.

Software and code

Policy information about availability of computer code

Data collection	No software was used.
Data analysis	PepSIRF v1.4.0, R version 4.2 (and custom code generated therein, available at https://osf.io/6ht43/), R clusterProfiler library v4.2.2, renv package 0.16.0.

For manuscripts utilizing custom algorithms or software that are central to the research but not yet described in published literature, software must be made available to editors and reviewers. We strongly encourage code deposition in a community repository (e.g. GitHub). See the Nature Portfolio guidelines for submitting code & software for further information.

Data

Policy information about availability of data

All manuscripts must include a data availability statement. This statement should provide the following information, where applicable:

- Accession codes, unique identifiers, or web links for publicly available datasets
- A description of any restrictions on data availability
- For clinical datasets or third party data, please ensure that the statement adheres to our policy

Data presented in this manuscript are available in the Open Science Forum (OSF) under DOI 10.17605/OSF.IO/6HT43, available at https://osf.io/6ht43/.

Human research participants

Policy information about studies involving human research participants and Sex and Gender in Research.

Reporting on sex and gender	Sex information was collected and is presented and analyzed in Supplementary Figures 4 and 8.
Population characteristics	The 'ACS cohort' consists of 12-18 year-old Mycobacterium Tuberculosis-infected participants residing in the Western Cape of South Africa. The 'SISCAPA' dataset is from a single 60+ year-old healthy male participant The 'MyImmunity cohort' comprises 30 healthy participants aged 18-58+. The 'COVID-19 vaccine' cohort consists of 21 healthy participants aged 18-60 + with no known history of SARS-CoV-2 exposure.
Recruitment	Recruitment for the 'ACS cohort' was performed in a prior study (described in reference #22). The study enrolled and followed participants who attended high schools in the town of Worcester, in the Western Cape of South Africa for a total of 24 months. The 'MyImmunity', 'SISCAPA' and 'COVID-19 vaccine' cohorts all comprise healthy adult volunteers from North America. They were recruited in connection with a research institution / assay company which may generally enrich for subjects who may be more educated and health-conscious than the general population average, and could impact the number and type of natural infection events in these latter cohorts.
Ethics oversight	Western IRB (WIRB) under #20191236.

Note that full information on the approval of the study protocol must also be provided in the manuscript.

Field-specific reporting

Please select the one below that is the best fit for your research. If you are not sure, read the appropriate sections before making your selection.

☒ Life sciences ☐ Behavioural & social sciences ☐ Ecological, evolutionary & environmental sciences

For a reference copy of the document with all sections, see nature.com/documents/nr-reporting-summary-flat.pdf

Life sciences study design

All studies must disclose on these points even when the disclosure is negative.

Sample size	Sample size (4 cohorts, the biggest of which was n=65 donors x 4 timepoints = 260 samples) was based on sample availability and represents the largest longitudinal highly-multiplexed serology study of which we are aware. Rather than participant-to-participant comparisons, our primary focus is intra-donor comparisons, which were powered by applying stringent statistical thresholds to the analysis of large datasets that include significant internal replication/redundancy (ie multiple peptides per epitope, multiple epitopes per species).
Data exclusions	No data were excluded.
Replication	Each dataset was performed once, using donor-level replicates to control the integrity of sample tracking, and peptide/epitope-level redundancy to boost confidence in the viral events called. We have also tested the reproducibility of our findings against orthogonal assays by running commercial ELISAs (Supplemental Figure 9).
Randomization	Randomization is not applicable to this study because there is no relevant intervention being studied.
Blinding	All viral profiles were generated and analyzed blinded to all covariates (age, sex, disease status) and then unblinded for final comparisons at the end.

Reporting for specific materials, systems and methods

We require information from authors about some types of materials, experimental systems and methods used in many studies. Here, indicate whether each material, system or method listed is relevant to your study. If you are not sure if a list item applies to your research, read the appropriate section before selecting a response.

Materials & experimental systems

n/a	Involved in the study
☒	☐ Antibodies
☒	☐ Eukaryotic cell lines
☒	☐ Palaeontology and archaeology
☒	☐ Animals and other organisms
☒	☐ Clinical data
☒	☐ Dual use research of concern

Methods

n/a	Involved in the study
☒	☐ ChIP-seq
☒	☐ Flow cytometry
☒	☐ MRI-based neuroimaging
